# Bilateral parapelvic cysts that mimic hydronephrosis in two imaging modalities: a case report

**DOI:** 10.1186/1757-1626-1-161

**Published:** 2008-09-18

**Authors:** Mohammad Kazem Tarzamni, Narges Sobhani, Nariman Nezami, Faramarz Ghiasi

**Affiliations:** 1 Department of Radiology, Tabriz University of Medical Sciences, Tabriz, Iran; 2 Young Researchers Club, Tabriz Islamic Azad University, Tabriz, Iran; 3 Drug Applied Research Center, Tabriz University of Medical Sciences, Tabriz, Iran

## Abstract

Parapelvic cysts are uncommon conditions that are usually found during autopsy. Their ultrasonographic appearance is similar to hydronephrosis. We report the case of a 46-year-old female with a 4-year history of vague flank pain and a previous history of bilateral moderate hydronephrosis. The patient was investigated by ultrasonography and non-enhanced CT scan, and finally diagnosed as bilateral parapelvic cysts by a contrast-enhanced CT scan. For any patient with hydronephrosis detected by sonography, the possibility of parapelvic cysts should be kept in mind, especially if no underlying cause is detected and other routine imaging is inconsistent with hydronephrosis. In such circumstances a CT scan with contrast enhancement should not be refused, and relying on sonographic signs, previously mentioned in literatures, can be misleading.

## Introduction

Parapelvic cysts are found in approximately 1.25–1.50% of autopsy cases [1]. Unlike simple renal cysts, they do not lie within the renal parenchyma. They are located on, or probably originate in, the hilus of the kidney in close proximity to the pelvis and major calyces [1]. They are thought to be lymphatic in origin and may be congenital [2]. Their appearance in an intravenous urogram (IVU) is similar to that of renal sinus lipomatosis and in sonography it is similar to hydronephrosis. Some signs have previously been described to help differentiate these cysts from hydronephrosis in ultrasound imaging. We present a case of bilateral parapelvic cysts in which conventional sonographic criteria could not help to make diagnosis.

## Case presentation

A 46-year-old female with a 4-year history of moderate bilateral hydronephrosis was referred by urologist to our centre for further evaluation. The patient complained of vague flank pain. Medical records showed that she had a normal IVU report but also multiple sonographies indicating bilateral hydronephrosis. Her previous physician referred her to us to resolve this apparent incongruity.

**Figure 1:**
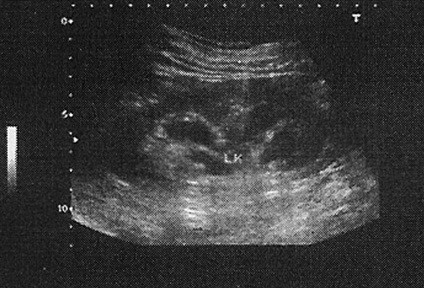
Hydronephrosis appearance in sonography.

On physical examination, no positive finding related to her history was found. Kidney sonography revealed dilated pelvises (Figure 1). In view of her history we suspected parapelvic cysts, but sonography did not show the cysts directly, and indirect signs such as the "convexity sign" did not help us to differentiate.

**Figure 2:**
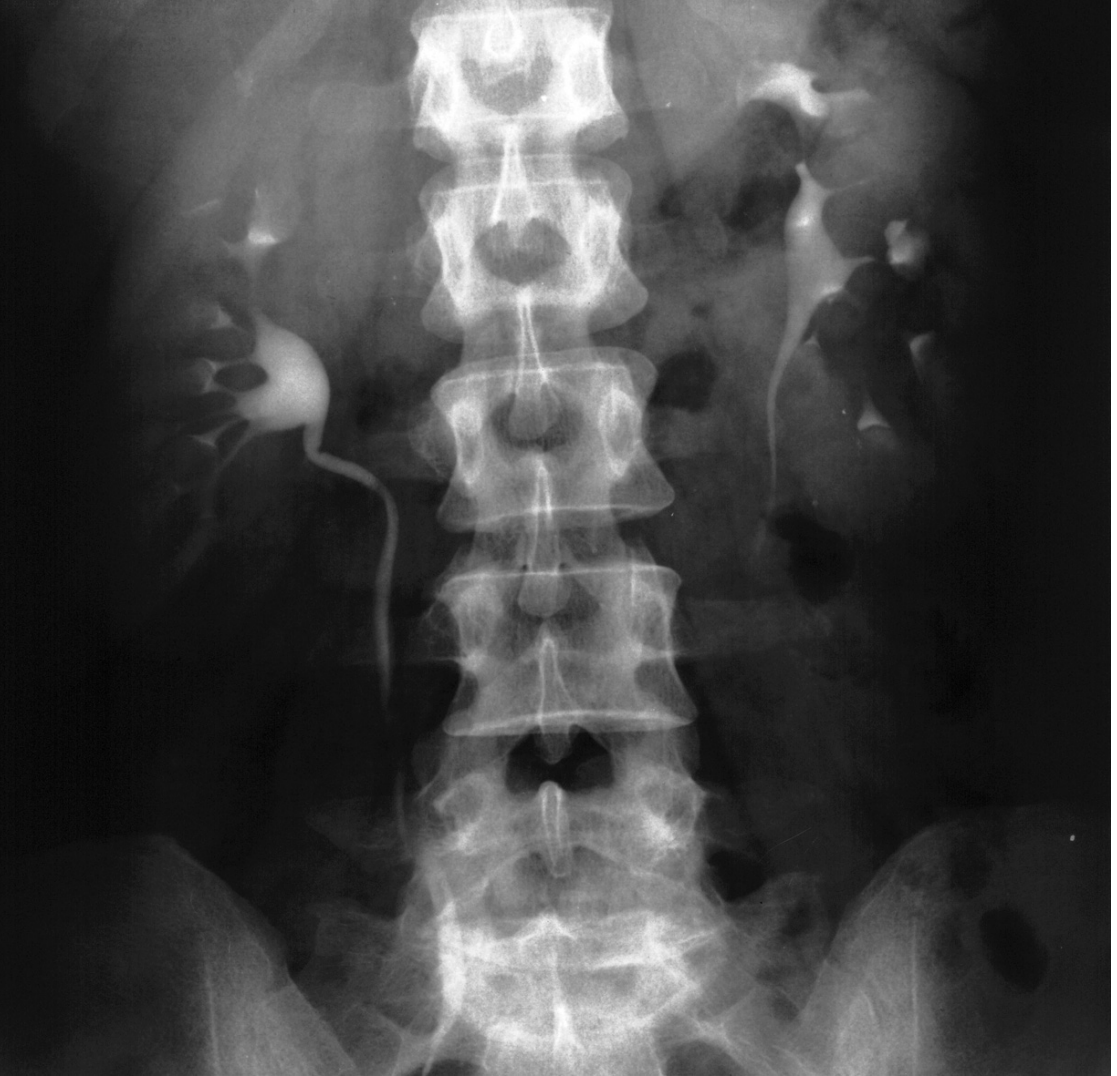
Stretching of calyces seen at IVU.

**Figure 3:**
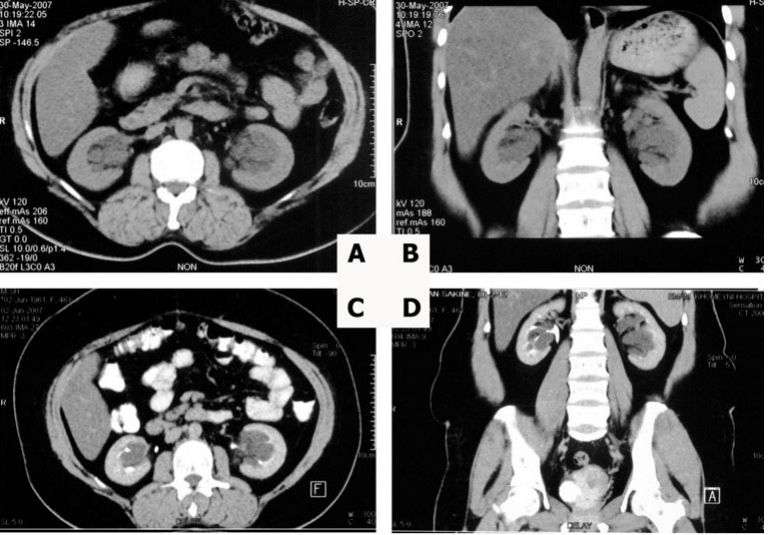
**Dilated pelvises seems to be due to hydronephrosis in a non-enhanced CT scan in axial (A) and sagittal reconstruction (B); only one separate parapelvic cyst is detected with this modality. Enhancement of parapelvic cysts septa and infundibuli after contrast injection in axial (C) and sagittal reconstruction (D).**

IVU also was performed and showed normal-looking fornices but stretched infundibuli (Figure 2). Then abdominal CT scans with and without contrast were implemented. The non-enhanced CT scan demonstrated dilated calyces and pelvises bilaterally, and no septa were observable (Figure 3A, B). Surprisingly, the CT scan with contrast showed multiple, bilateral parapelvic cysts (Figure 3C, D).

## Discussion

The terms peripelvic and parapelvic generally describe cysts around the renal pelvis or renal sinus [3]. In practice, both groups of cysts are often referred to as parapelvic cysts, and the term seems perfectly justifiable [2]. Parapelvic cysts do not communicate with the collecting system and are probably lymphatic in origin or develop from embryonic remnants. Most are asymptomatic, though they may cause hematuria, hypertension, hydronephrosis, or become infected [3,4]. In the present case, urine analysis was normal and the only complaint was vague flank pain. One etiological theory suggests that lymphatic cysts are secondary to obstruction. These are multiple and often bilateral [5]. Parapelvic cysts demonstrate stretching and compression of the calyces on IVU, similar to the appearance with marked renal sinus lipomatosis. On ultrasound they have the typical appearance of centrally-placed cysts, but may be mistaken for hydronephrosis [2,6]. When hydronephrosis is present, the anechoic fluid-filled calyces and renal pelvis can be seen to communicate, whereas multiple parapelvic cysts often have haphazard orientation and are seen as non-communicating renal sinus cystic masses [7,8]. In the present case the parapelvic cysts were too numerous – indeed, the pelvis was actually full of cysts – so there was no room for them to be oriented irregularly and therefore the orientation did not help us. Also, the cyst walls were too small to be detected separately by ultrasound.

A dilated renal pelvis may present as a cauliflower appearance, whereas a parapelvic cyst is more spherical in shape [9]. In our case, the multiple parapelvic cysts pushed each calyx from both sides and an echo-free space seemed to be continuous with each calyx, mimicking the cauliflower appearance.

Apart from points that could help us to differentiate parapelvic cysts from hydronephrosis, one study described a "convexity" sign that can be useful in making this distinction. Thus, cysts exhibit convex walls and curved outlines, whereas in hydronephrosis the walls of the dilated calyces are linear [6,10]. In our present case, the cysts were positioned back to back and were too crowded to be identified separately. Probably it was their crowded architecture that precluded ready identification. Because the pelvises were full of cysts, all the calyces were pushed from both sides, so the signs previously described in the literature such as the "convexity" sign were not diagnostically helpful; the cysts left no space for the calyceal walls to become convex.

Finally, a CT scan with contrast medium solved the problem. This emphasizes the point that in any patient with hydronephrosis detected by sonography, the possibility of parapelvic cysts should be kept in mind, especially if no underlying cause is detected and other routine imaging is inconsistent with hydronephrosis. In such circumstances, a CT scan with contrast can solve the problem. It means that trusting conventionally only on ultrasonographic and IVU findings can be misleading.

## Consent

Written informed consent was obtained from the patient for publication of this case report and any accompanying images.

## Competing interests

The authors declare that they have no competing interests.

## Authors' contributions

All authors made equal contributions. All authors read and approved the final manuscript.
